# Quasi-continuous parallel online scattered light, fluorescence and dissolved oxygen tension measurement combined with monitoring of the oxygen transfer rate in each well of a shaken microtiter plate

**DOI:** 10.1186/s12934-016-0608-2

**Published:** 2016-12-03

**Authors:** Tobias Ladner, Markus Held, David Flitsch, Mario Beckers, Jochen Büchs

**Affiliations:** AVT-Aachener Verfahrenstechnik, Biochemical Engineering, RWTH Aachen University, Worringerweg 1, 52074 Aachen, Germany

**Keywords:** BioLector, High-throughput screening, Online monitoring, Optical measurement, Oxygen transfer rate, k_L_a, Microtiter plate, µRAMOS

## Abstract

**Background:**

Microtiter plates (MTP) are often applied as culture vessels in high-throughput screening programs. If online measuring techniques are available, MTPs can also be applied in the first steps of process development. For such small-scale bioreactors dipping probes are usually too large; therefore, optical measurements are often used. For example, the BioLector technology allows for the online monitoring of scattered light and fluorescence in each well of a continuously orbitally shaken MTP. Although this system provides valuable data, these measurements are mainly of a semi-quantitative nature. Therefore, signal calibration is required to obtain absolute values. With the µRAMOS technology it became possible for the first time to quantify the oxygen transfer rate (OTR) separately in each well of an MTP. In this work, a device is presented that combines both techniques, to provide a hitherto unparalleled high amount of information from each single well.

**Results:**

Because both systems (BioLector and µRAMOS) are based on optical measurements, the measurements need to be synchronized to avoid interferences with the optical signals. The new experimental setup was applied for online monitoring in cultures of *Escherichia coli* and *Hansenula polymorpha*. It has been demonstrated that the well-to-well reproducibility is very high, and that the monitored signals provide reliable and valuable information about the process. With varying filling volumes, different maximum oxygen transfer capacities (OTR_max_) were adjusted in oxygen-limited cultures. The different degrees of stress during the culture due to oxygen limitation affected microbial growth and also impacted reproducibility from culture to culture. Furthermore, it was demonstrated that this new device significantly simplifies the experimental efforts: instead of parallel cultures in a shake flask and MTP, just one single experiment in MTP needs to be conducted to measure the OTR, dissolved oxygen tension (DOT), scattered light and fluorescence.

**Conclusions:**

The new device is a very suitable system for the online monitoring of cultures in continuously orbitally shaken MTPs. Due to the high number of parameters that can simultaneously be measured with this small-scale device, deeper insight into the investigated microbial system can be achieved. Furthermore, the experimental efforts to obtain OTR, DOT, scattered light and fluorescence signals during a culture are decreased. Ultimately, this new technology and the resulting high amount of collected data will eliminate the currently existing separation between screening and process development.

**Graphical abstract:**
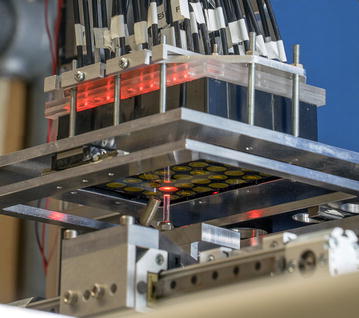
Picture of the combined μRAMOS and BioLector setup which allows for measurements of the oxygen transfer rate (OTR), dissolved oxygen tension (DOT), scattered light and fluorescence in each single well of an orbitally shaken microtiter plate.

**Electronic supplementary material:**

The online version of this article (doi:10.1186/s12934-016-0608-2) contains supplementary material, which is available to authorized users.

## Background

Microtiter plates (MTP) were developed in the 1950s and originally applied in serological studies [1]. However, soon their potential as culture vessel was identified and is more and more exploited within the recent past [1, 2]. MTPs offer the possibility to perform numerous cultures in parallel and, thus, accelerate process development [3, 4]. Furthermore, the costs per culture are significantly reduced due to decreased material consumption compared to large scale systems [4, 5].

To obtain a better understanding of the investigated processes, it is essential to have a reliable means for online monitoring. Hence, it is not surprising that many efforts have been made to get access to numerous process parameters also in MTPs [6–8]. Due to the miniaturization of these bioreactors, dipping probes are usually too large, and optical measurements need to be applied. The so called BioLector technology allows the online monitoring of each well in continuously orbitally shaken MTPs by means of fluorescence and scattered light [9, 10]. Because pH and the dissolved oxygen tension (DOT) are not directly measurable via fluorescence, fluorescence dyes sensitive towards protons or molecular oxygen were developed [11, 12]. These fluorescence dyes are commonly immobilized as sensor spots (optodes) at the bottom of each well [7, 8]. Recently, an alternative DOT measurement system based on dispersed infrared oxygen-sensitive nanoparticles was presented. It can be applied in any type of MTP without the need of optodes [13]. However, most of the so far presented online measurement techniques in MTPs only offer semi-qualitative data. The signals are usually presented as arbitrary units or as relative data requiring calibration. Nevertheless, to get also access to qualitative data in MTPs, parallel cultures in shake flasks were conducted and the results were considered as data received by one hypothetical bioreactor [14]. In a recent study, a so-called µRAMOS system was presented that allows for the direct online quantification of oxygen transfer rate (OTR) in each well [15]. µRAMOS is a miniaturized device adopted from the established RAMOS (Respiration Activity MOnitoring System) technique which allows online monitoring of eight parallel cultures in shake flask [16, 17].

In this study, the substantial potential of a device combining BioLector technology and the µRAMOS for online monitoring of cultures in MTPs is presented. By the simultaneous use of both monitoring techniques in one single MTP, increased information content about the biological growth and metabolic behavior can be obtained with very small experimental effort. Hence, it becomes possible to measure the following process parameters in each well: DOT, pH (with optodes), OTR, biomass (scattered light), tryptophan, NADH, riboflavin and, if applicable, protein levels via fluorescent proteins such as green fluorescent protein (GFP) derivatives and flavin-based fluorescent proteins (FbFP). The applicability is demonstrated in cultures of the bacterium *Escherichia coli* and the yeast *Hansenula polymorpha*. Furthermore, the impact of the filling volume on maximum oxygen transfer capacity (OTR_max_) and the resulting effects on biological growth are investigated. As for the first time an OTR and DOT signal is available in MTPs over time, the volumetric oxygen transfer coefficient (k_L_a) is calculated online during the cultures.

## Results and discussion

### Well-to-well reproducibility of *E. coli* BL21 (DE3) pRhotHi YFP cultures

**Fig. 1:**
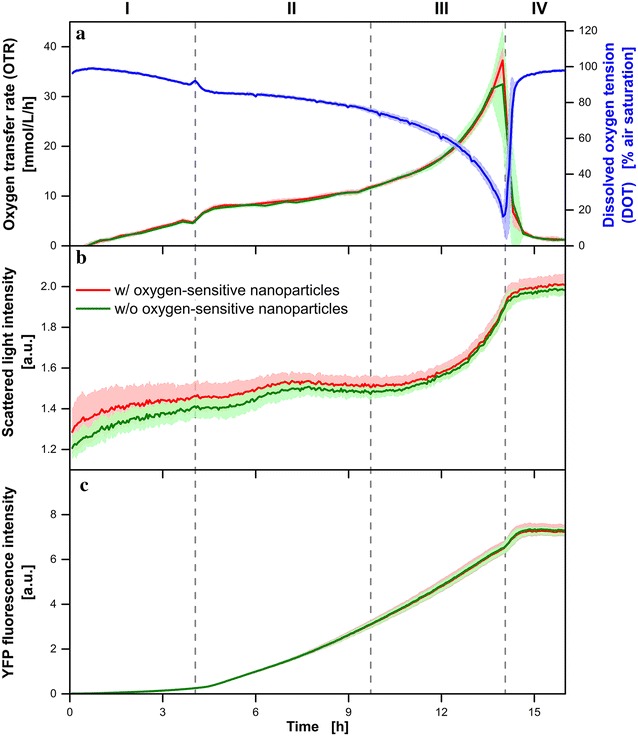
Cultivation of *E. coli* BL21 (DE3) pRhotHi YFP on synthetic Wilms-MOPS auto-induction medium containing 0.5 g L^−1^ glucose, 2 g L^−1^ lactose, 5 g L^−1^ glycerol and 0.2 M MOPS buffer with (w/) and without (w/o) 0.1 g L^−1^ dispersed infrared oxygen-sensitive nanoparticles. 20 parallel cultures with (*red*) and 22 parallel cultures without (*green*) 0.1 g L^−1^ oxygen-sensitive nanoparticles were performed in the same MTP. The presented signals are averages of parallel cultures. The corresponding standard deviations are represented by the surrounding *shaded areas*. **a** Online monitoring of the oxygen transfer rate (OTR). The dissolved oxygen tension (DOT) is only available for cultures where oxygen-sensitive nanoparticles were applied (*blue*). **b** Microbial growth monitored via scattered light at 650 nm. **c** Fluorescence intensity of YFP (λ_ex_ = 480 nm; λ_em_ = 522 nm). Cultivation conditions: 48 round deep-well MTP without optodes, V_L_ = 800 µL, n = 1000 rpm, shaking diameter d_0_ = 3 mm, 37 °C

The greatest benefit of high-throughput screening devices is the high number of parallel cultures that can be conducted within a single experiment. But, to allow a valid comparison of the performed cultures from well to well it is essential that cultures which are conducted under equal conditions proceed with the same manner. In Fig. 1 the well-to-well reproducibility of parallel *E. coli* cultures is investigated. In this model, the *E. coli* cells are cultivated on a synthetic Wilms-MOPS auto-induction medium that contains three initial carbon sources (0.5 g L^−1^ glucose, 2 g L^−1^ lactose and 5 g L^−1^ glycerol) [13, 14, 18]. All presented signals are averages of parallel cultures and the surrounding shaded areas indicate their corresponding standard deviations. For DOT measurements (blue, Fig. 1a), in 20 parallel cultures 0.1 g L^−1^ infrared oxygen-sensitive nanoparticles were additionally added to the culture broth. OTR (Fig. 1a), scattered light (Fig. 1b) and YFP fluorescence (Fig. 1c) of these cultivations containing oxygen-sensitive nanoparticles are depicted in red. In green, the signals of a group of 22 parallel cultures without nanoparticles are shown (Fig. 1a–c). Based on OTR and DOT (Fig. 1a), four typical phases, which are known for this kind of culture, can be distinguished (vertical dashed gray lines). Since the main focus of this section is the well-to-well reproducibility, a detailed analysis of the growth behavior is not carried out here. The growth behavior for this kind of culture is discussed in more detail in the next section for three *E. coli* clones, where a single amino acid was exchanged to create a recombinant protein.

During the entire culture, the OTR curves of both groups (with and without oxygen-sensitive nanoparticles) proceed in the same way (Fig. 1a). The small surrounding shaded areas (standard deviations) indicate that almost no differences in respiration activity occured within the parallel cultures. According to the literature [19], the fact that the dispersed oxygen-sensitive nanoparticles do not shown any effect on the breathing behavior indicates their biocompatibility and is in agreement with a previous study [13]. The DOT signal (blue, Fig. 1a) is only available for the parallel cultures containing oxygen-sensitive nanoparticles in the cultivation broth. As expected, the DOT appears as perfect mirror image of the OTR (Fig. 1a). According to the OTR signal, only a small surrounding shaded area is visible for the DOT measurement and, thus, just negligible differences between the measured DOTs within the parallel cultures were observed.

In the scattered light signal, a clear difference between both groups of parallel cultures (with and without oxygen-sensitive nanoparticles) was observed until 7 h of culture time (Fig. 1b). Due to the presence of dispersed infrared oxygen-sensitive nanoparticles, light scattering is increased (red, Fig. 1b) and higher signals compared to measurements without nanoparticles (green, Fig. 1b) are obtained. As indicated by the shrinking surrounding shaded areas, the standard deviation of the scattered light signals decreased over time. The underlying signal variations are known in literature and can be attributed to low biomass concentrations at the start of the culture [9]. When a critical biomass concentration is exceeded, a stable signal is obtained and the standard deviation is reduced (after approximately 7 h, Fig. 1b). At the end of the cultivation (IV: 14.2–16 h), almost the same signal is obtained for cultures with and without nanoparticles. Due to the microbial growth, higher biomass concentrations are reached, which results in increased light scattering. Thus, the overall impact of the dispersed nanoparticles on the light scattering becomes less significant at increased biomass concentrations.

Due to the induction by and consumption of lactose, this *E. coli* clone starts (after approximately 4 h, Fig. 1c) to synthesize yellow fluorescent proteins (YFP). In Fig. 1c, the production is clearly indicated by an increase of the YFP fluorescence signal intensity in phase II (4.7–11.7 h) and III (11.7–14.2 h). However, no differences between both groups of parallel cultures (with and without nanoparticles) were visible. Again, the small standard deviations (surrounding shaded areas) indicate an excellent reproducibility from well to well.

In the investigated parallel cultures of *E. coli* (Fig. 1) only small variations between parallel cultures were observed and, thus, the well-to-well reproducibility was very high. Consequently, the conditions (especially the air supply via the µRAMOS cover) in each well can be considered as equal. The OTR, DOT, scattered light and fluorescence signals of every well can be compared from well to well. It can be concluded that the dispersed infrared oxygen-sensitive nanoparticles (0.1 g L^−1^) only show a slight impact on the scattered light signal at low biomass concentrations and do not influence the microbial growth.

### Comparison of the respiration behavior of three *E. coli* BL21 (DE3) clones with single amino acid exchanges in a recombination protein

**Fig. 2:**
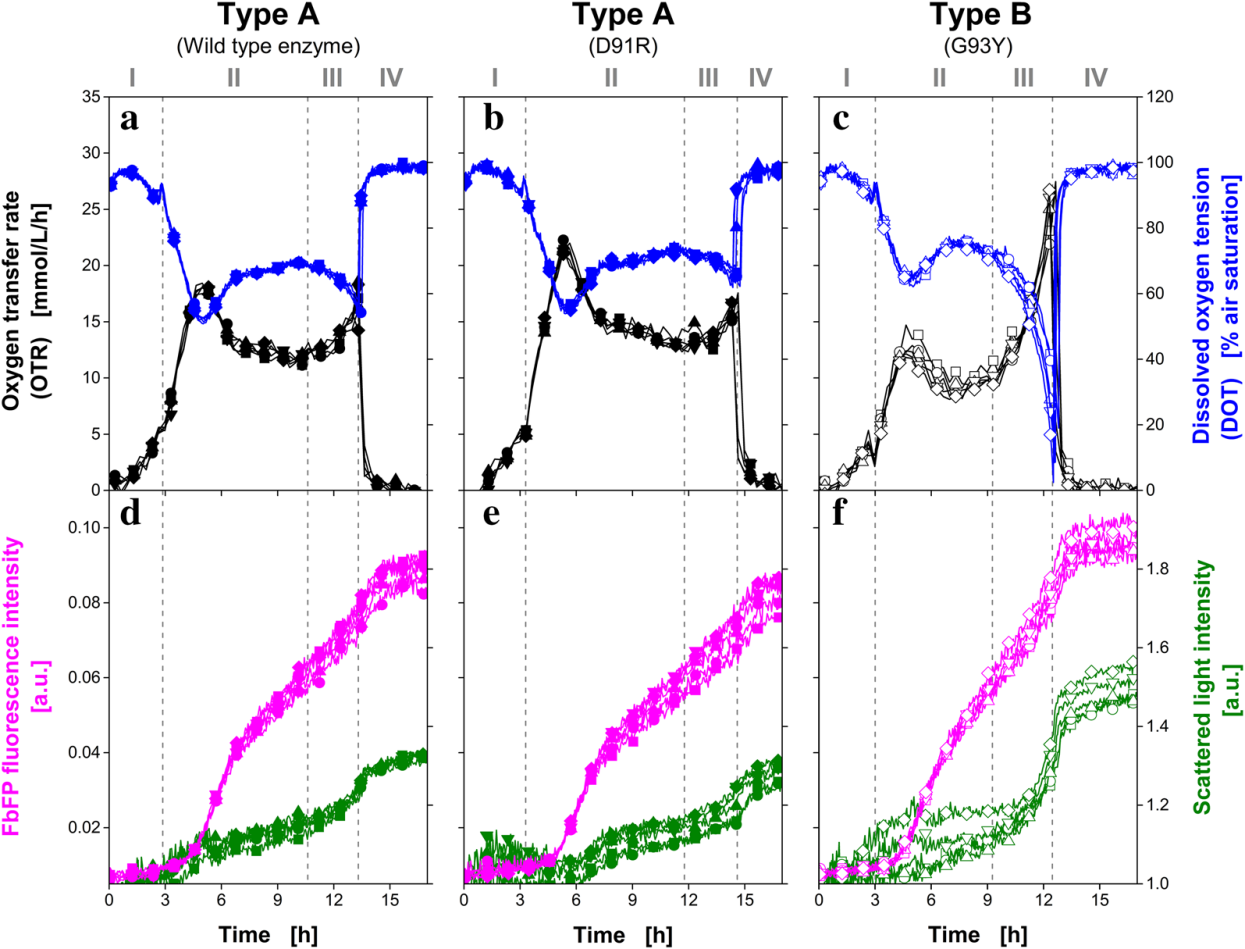
Comparison of *E. coli* BL21 (DE3) clones with single amino acid exchanges in a recombinant protein cultivated on synthetic Wilms-MOPS auto-induction medium containing 0.5 g L^−1^ glucose, 2 g L^−1^ lactose, 5 g L^−1^ glycerol and 0.2 M MOPS buffer. All cultures were performed in parallel in one MTP. Each group of cultures consists of five replicates. According to their respiration behavior the clones were divided in Type A (wild type enzyme, D91R) and Type B (G93Y). **a**–**c** Online monitoring of the oxygen transfer rate (OTR, *black lines*) and the dissolved oxygen tension (DOT, *blue lines*). The phases (*I–IV*) typical for this kind of culture are indicated by *dashed lines*: (*I*) increase in OTR due to growth on glucose, at the end of this phase glucose is exhausted; (*II*) product (FbFP) formation during parallel consumption of lactose and glycerol; (*III*) increase in OTR due to growth on residual glycerol; (*IV*) end of culture. **d**–**f** Microbial growth monitored via scattered light at 650 nm (*green lines*) and the fluorescence intensity of FbFP (λ_ex_ = 450 nm; λ_em_ = 492 nm). Cultivation conditions: 0.1 g L^−1^ infrared oxygen-sensitive nanoparticles; 48 round deep-well MTP without optodes, V_L_ = 800 µL, n = 1000 rpm, shaking diameter d_0_ = 3 mm, 37 °C

Rahmen et al. [18] investigated the influence of a single amino acid exchanges in a recombinant protein on the metabolic burden during protein production. In the mentioned work, microbial growth was monitored in parallel cultures with two devices: the RAMOS (shake flask) and BioLector (MTP). To provide perfectly comparable conditions for cultures in both scales, the engineering parameters have to be suitably adjusted and a large experimental effort is required. Due to the experimental setup presented here, the same information can be generated by means of one device with reduced and simplified experimental efforts in a single MTP experiment. Three *E. coli* BL21 (DE3) clones, which were already investigated by Rahmen et al. [18], were cultivated again and monitored with the new experimental setup. All clones were investigated in groups of parallel cultures consisting of five replicates. The same synthetic Wilms-MOPS auto-induction medium which was applied for the previous cultures (Fig. 1) was used. In the following, the three types of cultivation are discussed based on the online monitored signals (Fig. 2). The DOTs (blue, Fig. 2a–c) appear as mirror image of the OTRs (black, Fig. 2a–c) and the different phases of the cultivations can be identified based on these signals. As already mentioned above, for this kind of cultivation four typical phases can be distinguished. In Fig. 2, these phases are highlighted by vertical dashed lines. The course of the carbon source concentrations is given as supplementary data (Additional file 1: Figure S1) for *E. coli* clones belonging to respiration behavior Type A and Type B. In phase (I), growth on glucose occurs until its depletion, which is indicated by a short drop in OTR (Fig. 2a–c) and a corresponding small peak in the DOT (Fig. 2a–c). Then, parallel consumption of lactose and glycerol occurs (II). Within this phase, the respiration activity further increases and is then followed by a decrease of the OTR. The production of FbFP is induced due to the induction by and consumption of lactose (magenta, Fig. 2d–f). After depletion of lactose, the remaining glycerol is consumed in the subsequent phase (III). This leads to increased respiration activity and results in an OTR increase and corresponding DOT decrease. Phase (IV) is indicated by a decreasing respiration activity (OTR drops to 0 mmol L^−1^ h^−1^; DOT returns to 100% air saturation) due to the depletion of all carbon sources.

Rahmen et al. [18] classified the *E. coli* clones based on the respiration behavior in Types A and B. Clones, which are assigned to Type A reach an OTR of 15–20 mmol L^−1^ h^−1^ in phase (II) and (III), while the clones of respiration behavior Type B reach values below 15 mmol L^−1^ h^−1^ in phase (II) and an OTR of more than 25 mmol L^−1^ h^−1^ in phase (III) [18]. In accordance with this classification, the *E. coli* clone expressing the wild type enzyme (Fig. 2a, d) and D91R (Fig. 2b, e) belong to Type A, while G93Y (Fig. 2c, f) is part of Type B. These results fully agree with the findings presented by Rahmen et al. [18].

In Fig. 2d–f, protein formation (via FbFP fluorescence, magenta) and biomass concentration (via scattered light, green) over time are shown. With respect to the FbFP fluorescence signals (magenta, Fig. 2d–f), it becomes clear that the protein formation of all three *E. coli* clones starts between 4 and 6 h (phase II). Directly after the start of the protein production in phase (II), the strongest FbFP formation is indicated. Within the first three phases (I–III), merely slight scattered light (green, Fig. 2d–f) increases were observed which indicate that only small amounts of biomass are produced. Hence, the increased respiration activities (increased OTR) can be attributed to the metabolic activity due to protein formulation. After depletion of lactose, further growth on the remaining glycerol occurs (III). In this phase, the strongest biomass increase is indicated by the scattered light increase for all clones. Especially for G93Y (Type B, Fig. 2c, f) the exponential OTR increase indicates an undisturbed cell growth until glycerol depletion at the end of phase (III). In the final phase (IV) all carbon sources are depleted. Consequently, the respiration activity decreases and no further FbFP is synthetized.

Both *E. coli* clones belonging to respiration behavior Type A (wild-type enzyme, D91R) reached more or less the same level of scattered light at the end of the culture (green, Fig. 2d, e). Similar observations were made for the YFP fluorescence signals (magenta, Fig. 2d, e). In comparison with these *E. coli* clones, higher scattered light and YFP fluorescence intensities are obtained at the end of the culture of G93Y (Type B).

### Effect of different filling volumes on growth of *E. coli* BL21 (DE3) pRhotHi YFP

**Fig. 3:**
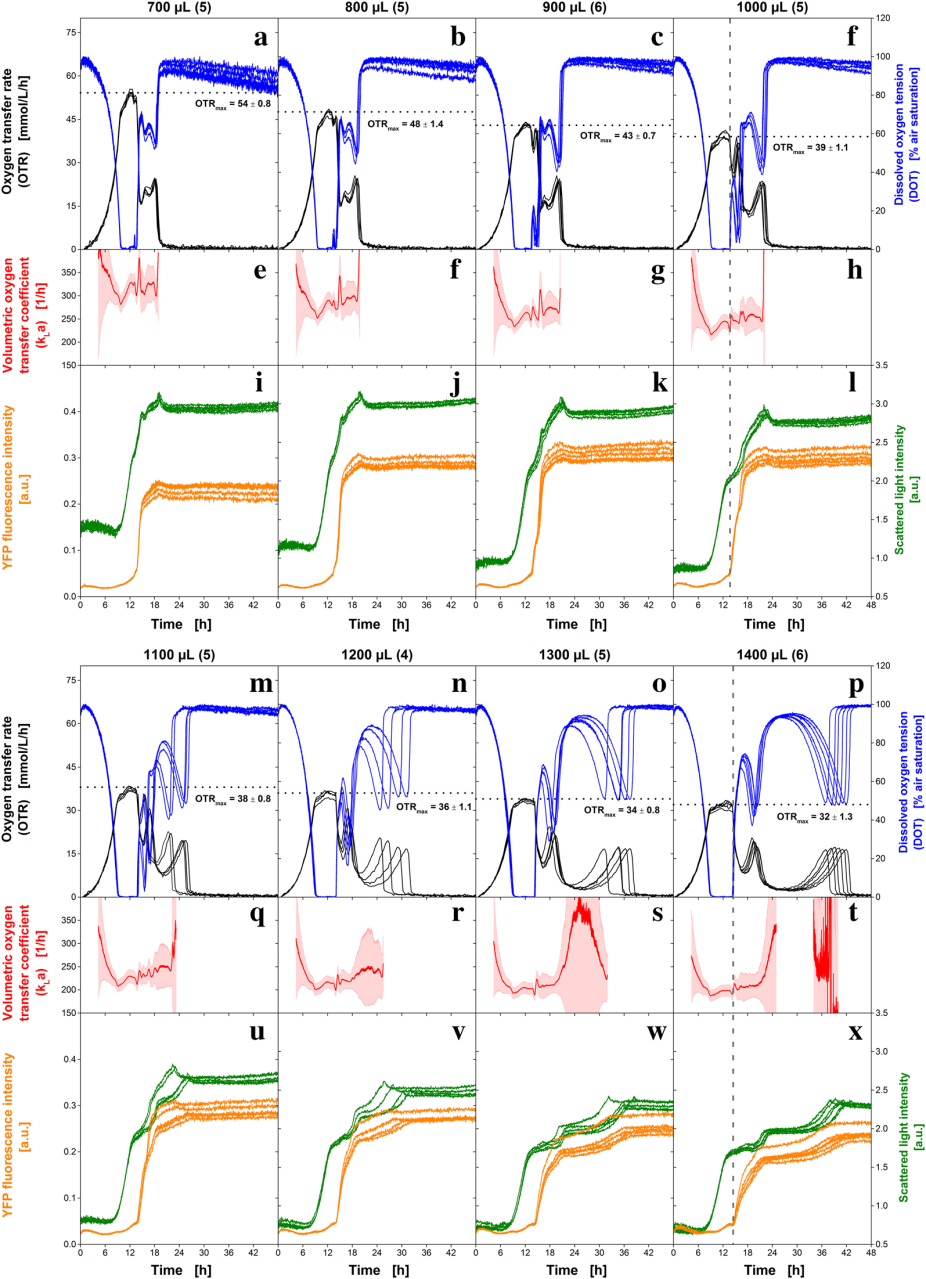
Effect of the filling volume on the growth of non-induced *E. coli* BL21 (DE3) pRhotHi YFP in synthetic Wilms-MOPS containing 20 g L^−1^ glucose and 0.2 M MOPS buffer. All cultures were performed in parallel in one MTP. In groups of parallel cultures with different filling volumes the oxygen transfer rate (OTR, *black lines*) and the dissolved oxygen tension (DOT, *blue lines*) (**a**–**d**, **m**–**p**) are monitored. The number of parallel cultures is given in *brackets* behind the filling volume. Also scattered light at 650 nm (*green lines*) and YFP fluorescence (λ_ex_ = 480 nm; λ_em_ = 522 nm, *orange lines*) (**i**–**l**, **u**–**x**) are presented. The averaged maximum oxygen transfer capacity (OTR_max_) is highlighted by a *black horizontal dotted line* for each filling volume. The volumetric oxygen transfer coefficient (k_L_a, *red lines*, Eq. 1) and the corresponding errors (*light red shaded area*, Eq. 2) were calculated (**e**–**h**, **q**–**t**) for OTR >5 mmol L^−1^ h^−1^ based on OTR and DOT. The end of oxygen limitation, which triggers the completion of YFP maturing, is indicated by a *gray vertical dashed line* for filling volumes of 1000 µL (**d**, **h**, **l**) and 1400 µL (**p**, **t**, **x**). Cultivation conditions: 0.1 g L^−1^ infrared oxygen-sensitive nanoparticles; 48 round deep-well MTP without optodes, n = 1000 rpm, shaking diameter d_0_ = 3 mm, 37 °C

The oxygen supply is one of the most critical process parameters in aerobic cultures. Especially in shake flasks and MTPs it is often challenging to provide sufficient oxygen supply during cultivation [20, 21]. Already in 1965, it was shown that oxygen-limited conditions during cultivation can affect microbial growth and product formation [22]. Lattermann et al. [23] presented an empirical correlation to estimate and compare the maximum oxygen transfer capacity (OTR_max_) in MTPs with different well shapes. Besides a relative perimeter, which considers different well geometries, the shaking frequency (n) and the filling volume (V_L_) are used in the empirical correlation for OTR_max_ assessments. Because all wells of a common 48 round deep-well MTP have the same shape and are equally affected by changes in shaking frequency, only the filling volume can be varied to achieve different OTR_max_ in one MTP experiment. In Fig. 3, parallel cultures of *E. coli* BL21 (DE3) pRhotHi YFP on synthetic Wilms-MOPS-Medium with 20 g L^−1^ glucose at a constant shaking frequency of 1000 rpm and different filling volumes are presented. All cultures were conducted in one single MTP experiment. The filling volume was varied from 700 to 1400 µL in steps of 100 µL, increasing from left to right in Fig. 3. For each group of cultures with equal filling volume, at least four cultures were conducted in parallel. The number of parallel cultures is given in brackets behind the filling volume. As indicated by DOT values of almost 0% air saturation (blue, Fig. 3a–d, m–p) all cultures are temporarily oxygen limited. During the oxygen limitation, OTR_max_ was calculated as average of the respective OTRs (black, Fig. 3a–d, m–p) and is illustrated as horizontal dotted lines for each filling volume.

In all cultures, exponential growth was observed until oxygen limited conditions occurred. That point is indicated in OTR by a characteristic horizontal plateau [16] (black, Fig. 3a–d, m–p) and in DOT by values close to 0% air saturation (blue, Fig. 3a–d, m–p). The general trend of decreasing OTR_max_ with increasing filling volume is in agreement with other studies [23, 24]. The oxygen limitation occurs earlier with increasing filling volumes (700 µL: 9.9 h; 1400 µL: 9.1 h). The duration of the oxygen limitation is directly correlated with the filling volume: the higher the filling volume the longer the oxygen limitation (700 µL: 3.7 h; 1400 µL: 5.4 h).

The pattern of the respective monitored signals is comparable for all cultures until the end of the oxygen limitation (Fig. 3). However, after the end of the oxygen limitation growth behavior varies from group to group. For filling volumes below 1000 µL it is quite difficult to differentiate whether only one or two further peaks in the OTR occur after the oxygen limitation (black, Fig. 3a–c). Since the DOT shows more or less a mirrored OTR signal, it also cannot be clarified based on the DOT measurements (blue, Fig. 3a–c). Even evaluating the scattered light and YFP fluorescence signal, no reliable conclusion can be drawn whether one or two metabolic phases occur after the end of the oxygen limitation (Fig. 3i–k). In contrast, for increased filling volumes (>1000 µL) it is evident that two phases of increased respiration activity are present after the oxygen limitation. OTR (black, Fig. 3d, m–p) and DOT (blue, Fig. 3d, m–p) show two clearly separated peaks after the oxygen limitation. These two phases of increased respiration activity can be attributed to the consumption of overflow metabolites, which are produced during the oxygen limitation. In literature it was already stated that overflow metabolites are increasingly formed during oxygen-limited conditions [25]. During culture of *E. coli* under oxygen-limited conditions often acetate was found as overflow metabolite, but also the formation of lactate and ethanol is reported [14, 25]. Via HPLC, offline concentrations of glucose, lactate, ethanol and acetate were measured over time and are provided as supplementary data for a filling volume of 1100 µL (Additional file 2: Figure S2). Since ethanol is not determined in detectable amounts during the culture, the phases of increased respiration activity can most likely be attributed to the consumption of lactate and acetate. With decreasing OTR_max_, extended total culture time is required to consume the overflow metabolites formed initially [25, 26]. In fact, the cultures with a filling volume of 700 µL are conducted within 20 h, while the last culture with 1400 µL is just completed after 43 h. Hence, the culture time is extended by 115%.

Furthermore, parallel cultures under similar operating conditions start to deviate significantly from each other after the end of the temporary oxygen limitation at filling volumes above 1000 µL (Fig. 3m–x). To confirm this observation all cultures were repeated and are shown in Additional file 3: Figure S3. Again, the parallel cultures start to deviate from each other after the end of the oxygen limitation at increased filling volumes. This interesting finding demonstrates that an insufficient oxygen supply significantly reduces the reproducibility of microbial growth and may explain increasing population heterogeneity. By means of single-cell microfluidic devices it became possible to analyze individual cells [2, 27, 28]. It was found that substantial cell-to-cell differences in physiological parameters (e.g. growth rate, resistance to stress) can occur and dramatically affect the microbial response on stress [29, 30]. The occurrences of such differences in the physiological parameters are stochastic processes [31]. Hence, the longer the stress remains, the higher is the probability that the response of the microorganisms on the perturbation deviates and an altered growth behavior occurs. Previous studies have shown that a prolongation of oxygen-limited conditions results in a stronger pH decrease since increased amounts of acidic overflow metabolites are produced [26]. Thus, besides the oxygen limitation also an acidification of the culture broth due to lactate and acetate formation induces perturbations. Both factors become larger with increasing filling volume. This ultimately results in a reduced reproducibility from culture to culture for filling volumes above 1000 µL (Fig. 3m–x).

In addition to OTR and DOT, YFP fluorescence and scattered light in each well were monitored online (Fig. 3i–l, u–x). In contrast to the cultivation on auto-induction medium (Fig. 1), an induction was not conducted in this culture. Still, changes of the YFP fluorescence signals (orange, Fig. 3i–l, u–x) are monitored over time due to leaky expression of this clone. The YFP fluorescence roughly appears to be a step function in all cultures. For all filling volumes, the YFP signals remains constant until the end of the oxygen limitation and then immediately bounces to a higher value. To make this interrelation clear, the end of the oxygen limitation is exemplarily highlighted by a vertical dashed line for filling volumes of 1000 µL (Fig. 3d, h, l) and 1400 µL (Fig. 3p, t, x). This phenomenon can be attributed to the fact that YFP, as a derivative of GFP, requires oxygen for maturing to fluorescence [32, 33]. Hence, the YFP which was produced during the oxygen limitation matures as soon as sufficient oxygen is present and the fluorescence signal abruptly increases.

The maximum measured YFP fluorescence intensities (after 48 h) were reached in cultures with a filling volume of 900 µL. This is so far not entirely unexpected, since it is known that controlled oxygen limitations can increase the productivity in some microbial systems [34, 35]. However, for this particular experiment this assumption would be misleading and result in wrong conclusions. In fluorescence measurements with the BioLector setup, to a certain extent also the filling volume has an impact. With increasing filling volume, the excitation light beam passes through an increasing amount of liquid. Thus, more fluorescent molecules in the liquid become excited than in a thin liquid layer (with the same concentration of fluorescent substance) and a higher fluorescence intensity is detected. Consequently, it is necessary to consider the different filling volumes to allow a comparison of the results. It was found that the YFP fluorescence intensities are non-linearly correlated to the filling volume. Therefore, calibration of the YFP fluorescence intensity for each specific filling volume is required. Due to the fact that the protein expression was not induced in these *E. coli* cultures, a detailed investigation of the maximum fluorescence intensity is not conducted in this work.

Similar YFP fluorescence, scattered light signal (green, Fig. 3i–l, u–x) correlated with filling volume. With increasing filling volume, the initial scattered light intensity is decreasing. This is in so far unfavorable, since the biomass concentration at the start is the same in each well and, thus, the same values should be detected. Such an offset in the scattered light signals can again be attributed to the optical measurement setup of the BioLector. A calibration for each filling volume would be required to compare the signals with each other. To obtain a relative value, the initial intensity can be subtracted from each measurement (I−I_0_). However, by applying this subtraction differences between initial biomass concentrations are no longer detectable. Nevertheless, already the pattern of the scattered light signal offers valuable information about the progress of the culture. During the first 7 h, the signals remain on a constant level and start slightly to increase within the oxygen-unlimited exponential growth. Under oxygen-limited conditions the scattered light signals show a linear increase for all filling volumes. The end of oxygen-limited conditions is indicated by a short stagnation of the scattered light signals (700–900 µL) or a further increase with a significantly reduced slope (1000–1400 µL). The succeeding phases of growth on overflow metabolites (lactate, acetate) are indicated by changes of the slope of scattered light and fit well to the OTR (black, Fig. 3) and DOT (blue, Fig. 3). At the end of the culture, the scattered light signals remain at a constant but slightly different level for each filling volume.

**Fig. 4:**
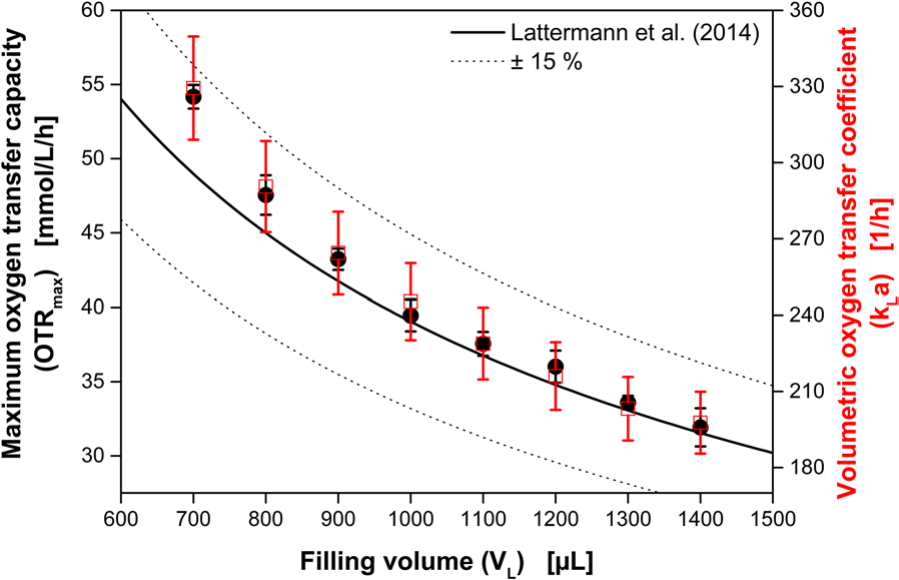
Dependency of the OTR_max_ (*black circles*) and k_L_a (*red squares*) on the filling volume for *E. coli* BL21 (DE3) pRhotHi YFP cultures in 48 round deep-well MTPs at 1000 rpm. Each measurement point is the average of minimum four cultures (compare Fig. 3). The standard deviation is presented by the corresponding *errors bars*. For comparison, in *black* the OTR_max_ is calculated based on an empirical correlation form literature [23]. The *black dashed lines* represent an error range of ±15% for the OTR_max_ calculated according to the empirical correlation. Cultivation conditions: synthetic Wilms-MOPS medium with 20 g L^−1^ glucose and 0.2 M MOPS buffer; 0.1 g L^−1^ infrared oxygen-sensitive nanoparticles; 48 round deep-well MTP without optodes, shaking diameter d_0_ = 3 mm, 37 °C

To confirm the OTR_max_ measured during the cultures, the values are compared in Fig. 4 with an empirical correlation which was presented by Lattermann et al. [23]. The empirical correlation [23] is illustrated as black line and the dashed black lines represent an error range of ±15%. For each filling volume, the maximum measured OTR values (≙OTR_max_) for parallel cultures were averaged. These are given as black circles, and their corresponding standard deviations are represented by error bars. It is obvious, that all measured OTR_max_ values are in very good agreement with the empirical correlation. Furthermore, the exponential increase of OTR_max_ with decreasing filling volume is recovered. In Table 1, the number of parallel cultures, the measured OTR_max_ and the OTR_max_ calculated based on the empirical correlation by Lattermann et al. [23] (OTR_max,Lattermann_) are summarized for all filling volumes. Additionally, the volumetric oxygen transfer coefficients (k_L_a) are given in Table 1. Since OTR (black, Fig. 3) and DOT measurements (blue, Fig. 3) were conducted during the cultures, it is possible to calculate the k_L_a according to Eq. 1 over time. The averaged k_L_a values of the parallel cultures are depicted as red lines. Their corresponding systematic k_L_a errors (Eq. 2) are shown by the surrounding shaded area in Fig. 3e–h, q–t. Due to the fact that the k_L_a estimation is fraught with high systematic errors at low respiration activities [36], the estimated k_L_a is very noisy at OTR values below 5 mmol L^−1^ h^−1^. Accordingly, the most reliable k_L_a estimation is conducted during oxygen-limited conditions. In Fig. 4, the estimated k_L_a values with the smallest systematic error (indicated by error bars) during the oxygen limitation are presented as red squares (right y–axis). As already found for the OTR_max_, an exponential increase of the k_L_a-value with decreasing filling volume is detected.

**Table 1:** Measured OTR_max_ and maximum oxygen transfer capacities calculated according to an empirical correlation (OTR_max,Lattermann_) as well as calculated k_L_a values during *E. coli* cultivations on synthetic Wilms-MOPS medium in 48 deep-well MTPs

Filling volume (µL)	Number of cultivations (−)	OTR_max_ (mmol L^−1^ h^−1^)	OTR_max,Lattermann_ (mmol L^−1^ h^−1^)	k_L_a (h^−1^)
700	5	54 ± 0.8	49 ± 7.3	329 ± 20
800	5	48 ± 1.4	45 ± 6.7	291 ± 18
900	6	43 ± 0.7	42 ± 6.3	264 ± 16
1000	5	39 ± 1.1	39 ± 5.9	245 ± 15
1100	5	38 ± 0.8	37 ± 5.5	229 ± 14
1200	4	36 ± 1.1	35 ± 5.2	216 ± 13
1300	5	34 ± 0.5	33 ± 5.0	203 ± 13
1400	6	32 ± 1.3	32 ± 4.7	198 ± 12

### Comparison of *H. polymorpha* RB11 pC10-*FMD* (P_*FMD*_-*GFP*) cultures in synthetic Syn-6-MES medium with different magnesium concentrations

**Fig. 5:**
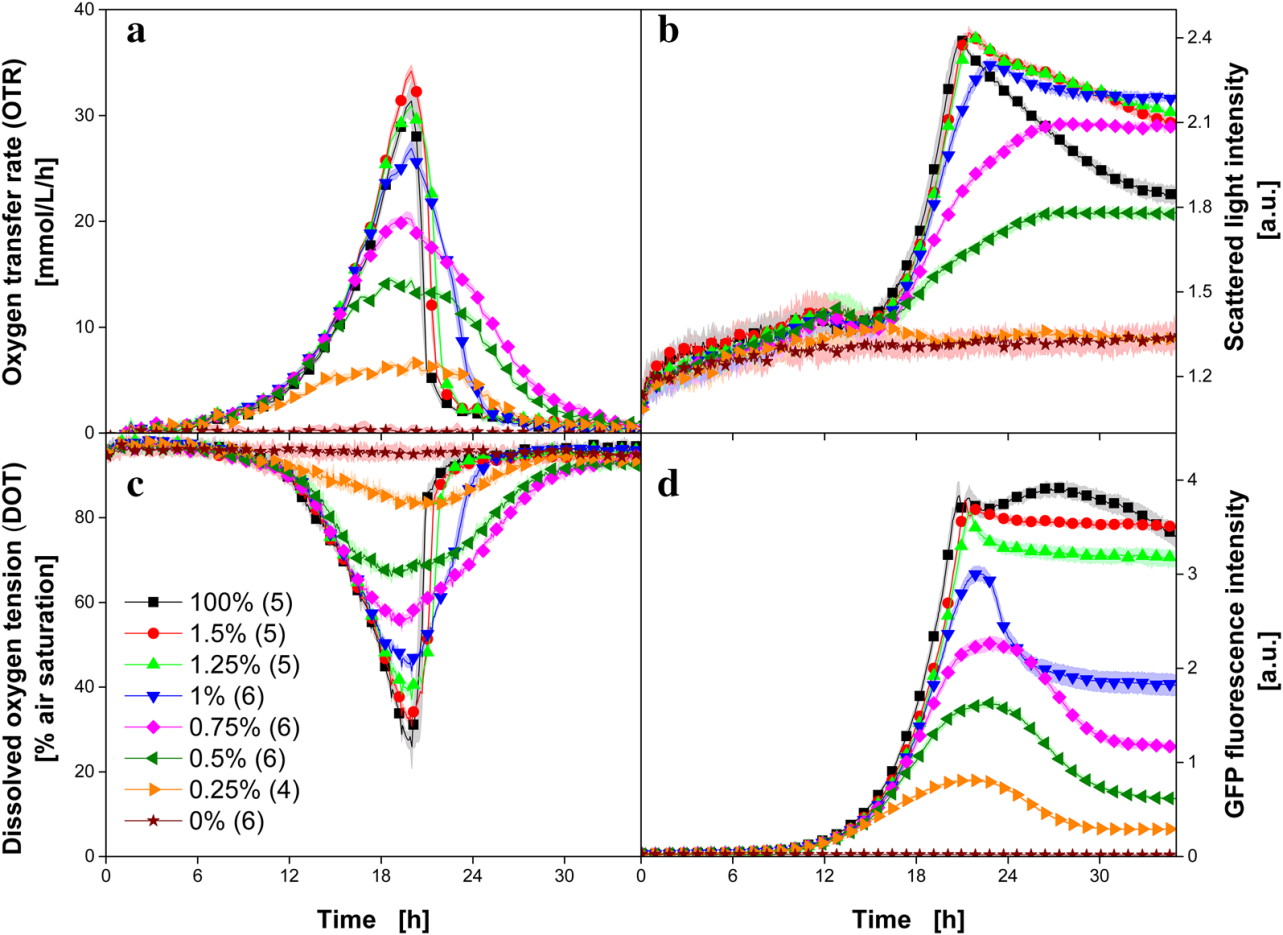
Comparison of *H. polymorpha* RB11 pC10-*FMD* (P_*FMD*_-*GFP*) cultures in synthetic Syn-6-MES medium containing 10 g L^−1^ glycerol and 0.12 M MES buffer with different concentrations of magnesium. The percentages refer to 3 g L^−1^ MgSO_4_·7H_2_O which is contained in the original medium. The presented signals are averages of parallel cultures in one MTP. The corresponding standard deviations are represented by the surrounding *shaded areas*. The number of investigated parallel cultures is specified in *brackets* in the legend behind the percentages. **a** Oxygen transfer rate (OTR). **b** Scattered light intensity at 650 nm. **c** Dissolved oxygen tension (DOT) measured via infrared oxygen-sensitive nanoparticles. **d** Fluorescence intensity of GFP (λ_ex_ = 510 nm; λ_em_ = 532 nm). Cultivation conditions: 0.1 g L^−1^ infrared oxygen-sensitive nanoparticles; 48 round deep-well MTP without optodes, V_L_ = 800 µL, n = 1000 rpm, shaking diameter d_0_ = 3 mm, 30 °C

In a fourth example, the methylotrophic yeast *H. polymorpha* was cultivated on synthetic Syn-6-MES medium with 10 g L^−1^ glucose and different amounts of magnesium. This culture scheme was already investigated by Kottmeier et al. [37] for the effect of a secondary substrate limitation (magnesium limitation) on biomass and product formation. Therefore, Kottmeier et al. [37] performed several parallel cultures in a BioLector (MTP) and a RAMOS device (shake flask). Because both techniques are combined within the new experimental setup, the same information can be obtained directly in parallel cultures within a single MTP experiment. In Fig. 5, the signals of OTR (Fig. 5a), scattered light (Fig. 5b), DOT (Fig. 5c) and the fluorescence intensity of green fluorescent protein (GFP) (Fig. 5d) are depicted over time. In all figures the same color code is used to indicate the percentage of magnesium in the culture broth. All cultures were performed as replicates with a minimum of four cultivations and the number of parallel conducted cultures is given in brackets behind the percentage in the figure legend (Fig. 5c). The presented signals are averaged values of the corresponding cultures. Their standard deviations are illustrated by the surrounding shaded areas. Based on the low standard deviations it can again be stated that the parallel cultures excellently match and proceed in the same manner.

At first glance, one notices that OTR (Fig. 5a) and DOT (Fig. 5c) appear as perfect mirror images of each other. Except the cultures without magnesium (0%) all cultures start growing on glucose with the same growth rate. For all cultures containing magnesium, exponential growth is indicated in the OTR (Fig. 5a) during the first 13 h. Due to the consumption of the dissolved oxygen in the culture broth, a corresponding exponential decrease appears in the DOT (Fig. 5c). The OTR of the reference cultures with 100% magnesium shows the typical pattern of unlimited growth [16]. For the cultures with 1.25, 1.5 and 100% magnesium, no significant differences in DOT and OTR are observed. However, the OTR (Fig. 5a) and the DOT (Fig. 5c) of the cultures containing less than 1.25% magnesium of the original medium deviate from the reference cultures and, thus, indicate a growth limitation by magnesium. After 13 h these cultures terminate the exponential OTR increase, transit through a maximum at 19 h (e.g. 6.5 mmol L^−1^ h^−1^ for the culture with 0.25% magnesium) and then return to 0 mmol L^−1^ h^−1^ after approximately 28 h. None of these cultures reached the same maximum OTR value as the reference (100%). With increasing magnesium limitation, the absolute maximum of the OTR decreased (Fig. 5a). The patterns of the OTR curves for 0.25–1% magnesium are quite similar to each other: in contrast to a sharp drop after 20 h like found in the reference culture (100%), a broad peak is detected. While the sharp OTR drop in the cultures with 1.25, 1.5 and 100% magnesium is explained by the instant depletion of glycerol, the end of the cultures for the other cultures is slowed down by the magnesium depletion. This characteristic pattern of the OTR indicates an substrate limitation expect the carbon source [16]. The integrals of OTR correspond to the area below the respective OTR curve and, thus, represent the total amount of consumed oxygen. Due to the fact that the integrals of the cultures with magnesium concentrations below 1.25% are significantly smaller than the integrals of the reference cultures (Fig. 5a), it can be concluded that only part of the provided total amount of glycerol was consumed [37]. Kottmeier et al. [37] also presented cultures in potassium- and phosphate-limited media. In these cultures, the total amount of provided glycerol was consumed, but significantly slower than in unlimited media. Because the focus of the present study is the introduction of the newly developed measurement system, a detailed investigation of changes in the cell physiology due to magnesium depletion is not conducted here.

In Fig. 5b, scattered light over time is presented. The signals of the cultures without magnesium (0%) remain on the same level during the entire cultures and, thus, indicate again that no microbial growth is present. Slightly increased scattered light signals are obtained for the cultures with 0.25% magnesium. However, based only on the scattered light it would not be possible to decide whether microbial growth occurred in these cultures or not. Just in combination with OTR (Fig. 5a) or DOT (Fig. 5c) the state of the cultures can be clarified. In all other cultures an increase of the scattered light signal is monitored after 15 h (Fig. 5b). The cultures containing 0.5% magnesium show a linear increase until 26 h and remain on a constant level for the rest of the culture. A similar pattern is found for the 0.75% cultures, but with a higher scattered light at the end of the culture (35 h). In contrast, the cultures with higher magnesium concentrations (1–100%) proceed with an exponential increase after 15 h. For 1.25, 1.5 and 100% comparable absolute maximum scattered light intensities (2.4 a.u.) are measured after 21 h. The point of time when the maximum scattered light is measured roughly agrees with the time of the maximum of the OTR (Fig. 5a) and indicates the depletion of glycerol. For the reference cultures (100%) a strong decrease of the scattered light is clearly visible. This decreasing trend of the scattered light signal is already known in literature and was attributed to morphological changes of microorganisms and the development of subpopulations [37, 38].

GFP fluorescence over time is presented in Fig. 5d. Since the FMD promoter is partly de-repressed during growth on glycerol, the GFP formation is more or less coupled with the biomass production [37, 39]. Since no growth was present for the 0% cultures, no GFP was produced. For all other cultures, the increase in fluorescence signal was monitored after approximately 10 h. The time when the first GFP is detected coincides with the increase of the respiration activity (Fig. 5a) and confirms that the GFP production is more or less coupled with the biomass formation in this strain. In general, the maximum GFP fluorescence during cultivation is increasing with increasing magnesium concentration.

## Conclusions

MTPs are often the bioreactors of choice for screening programs and in early stages of a process development. Especially, if numerous clones need to be investigated, the high number of in parallel conducted experiments is very advantageous. In this work, a new device for online monitoring in MTPs is presented. The system combines the benefits of BioLector [9] and µRAMOS technology [15]. Because all measurements are based on optical signals, a special measuring sequence had to be developed to avoid interference among various measurement systems. Information (OTR, DOT, scattered light and fluorescence), which was so far just available by parallel cultures in shake flasks and MTPs [14, 18], can now be obtained in one single MTP experiment. Hence, several process parameters are received from the same scale and the experimental efforts are significantly reduced. It is shown that a high well-to-well reproducibility in parallel cultures is reached with the new device (OTR_max_ variation below 3% during cultivation). The system showed its large potential for online monitoring throughout cultures of *E. coli* and *H. polymorpha*. These monitoring signals provide a better understanding of the investigated cultures. Due to varying filling volumes, different OTR_max_ were adjusted in one single MTP experiment and, thus, different degrees of oxygen limitation during the cultures were realized. In these experiments it was found that the reproducibility from culture to culture is significantly reduced if prolonged oxygen limitations are present in the culture. Furthermore, the k_L_a was calculated over time based on OTR and DOT, which were obtained during the cultures. The measurements and calculations turned out to be in full agreement with an empirical correlation found in literature [23]. Altogether, this new device appears as a promising parallel small-scale monitoring system for investigating cultures in MTPs. With little experimental effort, a hitherto unparalleled high amount of valuable information about the process is obtained.

Traditionally, screening (without online monitoring) and process development were clearly separate tasks, assigned to different research units in industry. With the introduced new technology differences between (secondary) screening and process development more or less disappear and a lot of information is already generated in the (secondary) screening programs. This will result in a secured selection of the most suitable strain in screening and an acceleration of process development.

## Methods

### Microorganisms

*Escherichia coli* BL21 (DE3) pRhotHi YFP expressing yellow fluorescence protein (YFP) and having a kanamycin resistance as well as *E. coli* BL21(DE3):pET22b(+)–His6-LOV-BSLA clones [18] (wild type enzyme: −wt, D91R: −Asp91Arg; G93Y: −Gly93Tyr) expressing a flavin-based fluorescent protein (FbFP) and characterized by ampicillin resistance were applied in this study. Furthermore, cultures with *H. polymorpha* RB11 pC10-*FMD* (P_*FMD*_-*GFP*) expressing the green fluorescent protein (GFP) were conducted. All strains were maintained in cryo stocks at −80 °C.

### Media and culture

For sterilization, all culture media were autoclaved (20 min, 121 °C, 1 bar), if not otherwise mentioned. Pre-cultures were carried out in 250 mL shake flasks with 10 mL filling volume at a shaking frequency of 350 rpm (shaking diameter d_0_ = 50 mm) and inoculated with a cryo-culture. The culture temperature for pre-cultures was adjusted according to the temperature which was set in the corresponding main culture. The main cultures were performed in BioLector constructed in-house and µRAMOS prototype which is described below (Measurement setup). The main cultures were inoculated with pre-cultures adjusting an initial OD_600_ of 0.1. In cultures with DOT measurement, 0.1 g L^−1^ dispersed oxygen-sensitive nanoparticles were added to the culture medium.

The *E. coli* cultures were performed in synthetic Wilms-MOPS medium with 20 g L^−1^ glucose and 0.2 M MOPS buffer according to Scheidle et al. [40] or in modified synthetic Wilms-MOPS auto-induction medium containing 0.5 g L^−1^ glucose, 2 g L^−1^ lactose, 5 g L^−1^ glycerol and 0.2 M MOPS buffer according to Rahmen et al. [18]. For the latter, two pre-culture stages (complex and synthetic medium) were conducted as recommended [18].

*Hansenula polymorpha* RB11 pC10-*FMD* (P_*FMD*_-*GFP*) was cultivated in synthetic Syn6-MES medium according to Jeude et al. [41]. The pre-culture was washed with sterile sodium chloride solution (0.9% NaCl) before inoculation of the main culture to adjust the magnesium limitation as described by Kottmeier et al. [37].

### Measurement setup

**Fig. 6:**
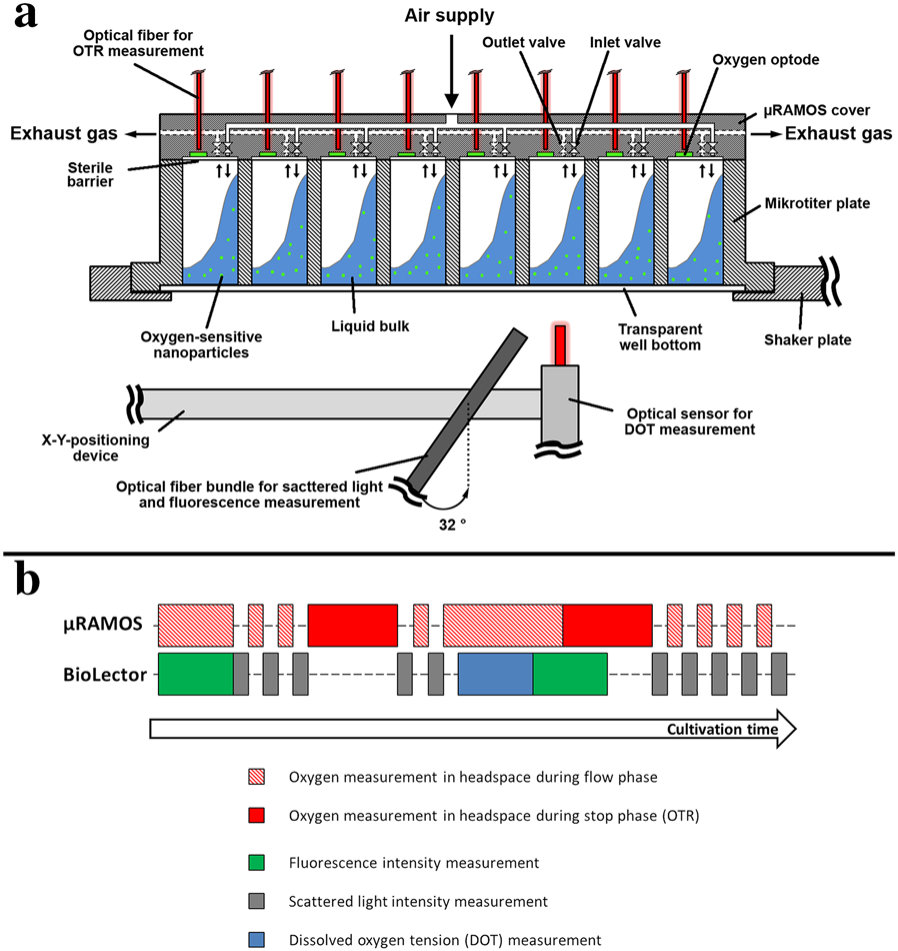
Measurement setup and operating principle. **a** Each well of the MTP is monitored via fluorescence, scattered light and dissolved oxygen tension (DOT) through the transparent bottom. By means of the µRAMOS device the oxygen concentration in the headspace of each well is monitored and the oxygen transfer rate (OTR) is determined. **b** The measurement phases of the µRAMOS and the BioLector device must be coordinated to avoid interferences between the optical measurements. Fluorescence and DOT measurements can be obtained simultaneously with measurements of the headspace oxygen concentration (for OTR evaluation) without interference. Scattered light and oxygen measurements in the gas phase (for OTR evaluation), can only be performed in alternating sequence

In Fig. 6a the experimental setup is illustrated and a picture is given as Additional file 4: Figure S4. A 48 round deep-well MTP (MTP-R48-B, m2p-labs GmbH, Baesweiler, Germany) is placed on the shaker table of an in-house constructed orbital shaking machine (shaking diameter d_0_ = 3 mm). For OTR measurements in each well, the µRAMOS cover was fixed on top of the MTP [15]. A sterile barrier (900371-T, HJ-Bioanalytik, Erkelenz, Germany) is clamped between µRAMOS cover and MTP to avoid contaminations (Fig. 6a). With the µRAMOS cover, equal gassing of each well is assured and the oxygen partial pressure in the headspace of each well is measured by an optical oxygen measurement system via optically isolated oxygen optodes. The optical oxygen measurement is based on fluorescence lifetime detection. Therefore, the excitation light is modulated in a sinus shape and, thus, the fluorescence emission shows an equal modulation. Based on the phase shift between excitation and emission, the oxygen partial pressure is calculated according to the Stern–Volmer equation. To allow simultaneous measurements for one whole row of wells (eight wells), different excitation light modulation frequencies (3550–4000 Hz) are applied in the optical multiplexer of the µRAMOS [15]. Thus, no interferences between neighboring oxygen sensors occur. For a precise determination of the OTR, the gassing of each well is interrupted by closing the microfluidic inlet and outlet valve (“stop-phase”). This OTR measurement method is adopted from the established RAMOS device for shake flasks [16, 17].

For fluorescence, scattered light and DOT measurements, an in-house constructed specific BioLector setup is placed below the MTP [9, 42]. Fluorescence, scattered light (λ = 650 nm) and DOT measurements are performed through the transparent MTP bottom. Fluorescence and scattered light are measured with a FluoroMax-4 spectrofluorometer (HORIBA Jobin Y, Munich, Germany), which is connected to a Y-shaped optical fiber bundle (UV–VIS, LEONI Fiber Optics GmbH, Neuhaus-Schierschnitz, Germany). The optical fiber bundle is mounted with an angle of 32° to the vertical center of the well (Fig. 6a). The DOT measurement is accomplished by means of an optical oxygen sensor (PICO2OEM sensor, Pyro Science GmbH, Aachen, Germany) in combination with dispersed infrared oxygen-sensitive nanoparticles (OXNANO, Pyro Science GmbH, Aachen, Germany). As suggested in literature [13], the optical oxygen sensor was equipped with an optical fiber rod of 3 mm diameter and positioned in vertical orientation (angle of 0° to the vertical center of the well) to reduce the required oxygen-sensitive nanoparticle concentration. To realize a sequential measurement of each well, one end of the Y-shaped optical fiber bundle and the optical oxygen sensor are displaced from well to well with an X–Y-positioning device (DGE-ZR, Festo AG & Co. KG, Esslingen, Germany).

Since the determination of all process parameters is based on optical measurements, the measurement sequence must be coordinated to avoid interferences between each measurement system. For all oxygen measurements (gas and liquid phase), the same optical measurement system with red light excitation (λ_ex_ = 620 nm) and NIR detection (λ_em_ = 760 nm) was applied. An exemplary sequence of the performed measurements is shown in Fig. 6b. It can be distinguished between measurements of the µRAMOS (above the MTP) and the BioLector (below the MTP). Measurements of the oxygen partial pressure in the headspace via µRAMOS and fluorescence measurements (BioLector) in the culture broth are simultaneously possible, as long as the investigated wavelength pairs deviate (adequately) from the wavelengths used for oxygen measurement (approximately 20 nm). Due to the applied optical isolation of the oxygen sensor spots (µRAMOS), also DOT measurements are simultaneously possible. In contrast, scattered light (BioLector) and oxygen measurements in the gas phase (µRAMOS) can only be performed alternately due to the risk of interferences. Within the measurement phase for OTR determination (“stop-phase”), a maximum high data density is advantageous to reduce the estimation error. Thus, the µRAMOS measurement is preferred within the “stop-phase” and the scattered light measurement has to wait (compare Fig. 6b). After the end of the “stop-phase”, the scattered light measurement is continued. The just described coordinated measurement sequence is repeated all time during the culture.

### k_L_a calculation

Based on the measurement of the oxygen partial pressure in the headspace of each well ($p_{{O_{2} }}^{gas}$) [bar], the determined OTR (mmol L^−1^ h^−1^) and the measured DOT (% air saturation), the k_L_a (h^−1^) is calculated during the µRAMOS aeration phases according to Eq. 1:

1 $${\text{k}}_{\text{L}} {\text{a}} = \frac{\text{OTR}}{{{\text{L}}_{{{\text{O}}_{2} }} \cdot \left( {{\text{p}}_{{{\text{O}}_{2} }}^{\text{gas}} - \frac{\text{DOT}}{100} \cdot {\text{p}}_{{{\text{O}}_{2} }}^{\text{cal}} } \right)}}$$

with $p_{{O_{2} }}^{cal}$ the headspace oxygen partial pressure during calibration (0.21 bar) and $L_{{O_{2} }}$ the oxygen solubility [mol L^−1^ bar^−1^], which was calculated according to literature [43–45]. Both values were considered to be constants during cultivation. To test the sensitivity of the k_L_a determination, a Gaussian error propagation was performed as described in the literature [36]. The results are given in Eq. 2.

2 $$\bf{\eqalign{ & \sigma _{{k_L}a}^2 = {\left( {\frac{1}{{{L_{{O_2}}} \cdot \left( {p_{{O_2}}^{gas} - \frac{{DOT}}{{100}} \cdot p_{{O_2}}^{cal}} \right)}}} \right)^2} \cdot \sigma _{OTR}^2 \cr & \qquad \quad + {\left( {\frac{{OTR}}{{{L_{{O_2}}} \cdot \left( {p_{{O_2}}^{gas} - \frac{{DOT}}{{100}} \cdot p_{{O_2}}^{cal}} \right)}}} \right)^2} \cdot \sigma _{P_{{O_2}}^{gas}}^2 \cr & \qquad \quad + {\left( {\frac{{OTR}}{{L_{{O_2}}^2 \cdot \left( {p_{{O_2}}^{gas} - \frac{{DOT}}{{100}} \cdot p_{{O_2}}^{cal}} \right)}}} \right)^2} \cdot \sigma _{{L_{{O_2}}}}^2 \cr & \qquad \quad + {\left( {\frac{{OTR \cdot \frac{{p_{{O_2}}^{cal}}}{{100}}}}{{L_{{O_2}}^2 \cdot {{\left( {p_{{O_2}}^{gas} - \frac{{DOT}}{{100}} \cdot p_{{O_2}}^{cal}} \right)}^2}}}} \right)^2} \cdot \sigma _{DOT}^2 \cr}}$$

${{\sigma }}_{\text{OTR}}$ (mmol L^−1^ h^−1^) is the assumed systematic error of the measured $OTR$ (5%), ${{\sigma }}_{{{\text{p}}_{{{\text{O}}_{2} }}^{\text{gas}} }}$ [bar] is the assumed systematic error of the measured $p_{{O_{2} }}^{gas}$ (3%), ${{\sigma }}_{{{\text{L}}_{{{\text{O}}_{2} }} }}$ (mol L^−1^ bar^−1^) is the assumed systematic error of the calculated $L_{{O_{2} }}$ (2%) and ${{\sigma }}_{\text{DOT}}$ (% air saturation) is the assumed systematic error of the measured $DOT$(3%).

## Abbreviations

DOT: dissolved oxygen tension (% air saturation); FbFP: Flavin-based fluorescent protein; GFP: green fluorescent protein; HPLC: high performance liquid chromatography; MTP: microtiter plate; OTR: oxygen transfer rate (mol L^−1^ h^−1^); OTR_max_: oxygen transfer rate (mol L^−1^ h^−1^); RAMOS: respiration activity monitoring system; YFP: yellow fluorescent protein.

### Symbols

d_0_: shaking diameter (mm); DOT: dissolved oxygen tension (% air saturation); k_L_a: volumetric oxygen transfer coefficient (h^−1^); L_O2_: oxygen solubility [mol L^−1^ bar^-1^ ]; n: shaking frequency (rpm); $p_{{O_{2} }}^{gas}$: oxygen partial pressure in gas phase (bar); $p_{{O_{2} }}^{cal}$: oxygen partial pressure in gas phase during calibration (bar); V_L_: liquid filling volume (mL); ${{\sigma }}_{\text{DOT}}$: assumed systematic error of ${\text{DOT}}$ estimation (% air saturation); ${{\sigma }}_{{{\text{k}}_{\text{L}} {\text{a}}}}$: error of kLa estimation (h^−1^); ${{\sigma }}_{{{\text{L}}_{{{\text{O}}_{2} }} }}$: assumed systematic error of ${\text{L}}_{{{\text{O}}_{2} }}$ estimation (mol L^−1^ bar^−1^); ${{\sigma }}_{\text{OTR}}$: assumed systematic error of ${\text{OTR}}$ estimation (mol L^−1^ bar^−1^); ${{\sigma }}_{{{\text{p}}_{{{\text{O}}_{2} }}^{\text{gas}} }}$: assumed systematic error of ${\text{p}}_{{{\text{O}}_{2} }}^{\text{gas}}$ estimation (bar).

## Additional files

**Additional file 1: Figure S1.** Comparison of *E. coli* BL21 (DE3) clones belonging to respiration behavior Type A (a, c, e) and Type B (b, d, f) cultivated on synthetic Wilms-MOPS auto-induction medium containing 0.5 g L^−1^ glucose, 2 g L^−1^ lactose, 5 g L^−1^ glycerol and 0.2 M MOPS buffer. The oxygen transfer rates (OTR) (a, b), concentrations of glycerol, lactose and glucose (c, d) as well as the FbFP fluorescence intensities (e, f) are presented over time. Four typical cultivation phases (I–IV) can be distinguished by means of the OTR curves and are highlighted by vertical dashed gray lines. Cultivation conditions: 37 °C, 250 mL flasks, filling volume 10 mL, shaking frequency 350 rpm, shaking diameter 50 mm (in RAMOS); 37 °C, 48-well Flowerplate, filling volume 1 mL, shaking frequency 1500 rpm, shaking diameter 3 mm (in BioLector). The presented data has already been published by Rahmen et al. [18].

**Additional file 2: Figure S2.** Culture of *E. coli* BL21 pRhotHi YFP under oxygen-limited conditions in synthetic Wilms-MOPS containing 20 g L^−1^ glucose and 0.2 M MOPS buffer with a filling volume (V_L_) of 1100 µL. The dissolved oxygen tension (DOT) was monitored by means of dispersed infrared oxygen-sensitive nanoparticles. pH, optical density (OD_600_), glucose, acetate and lactate concentrations were evaluated from offline analysis of parallel wells. Cultivation conditions: synthetic Wilms-MOPS medium with 20 g L^−1^ glucose and 0.2 M MOPS buffer; 48 round deep-well MTP without optodes, OD_600,start_ = 0.05, n = 1000 rpm, shaking diameter d_0_ = 3 mm, 37 °C.

**Additional file 3: Figure S3.** Effect of the filling volume on the growth of non-induced *E.* *coli* BL21 (DE3) pRhotHi YFP in synthetic Wilms-MOPS containing 20 g L^−1^ glucose and 0.2 M MOPS buffer. These cultures are repetitions of the cultures presented in Fig. 4. All cultures were performed in parallel in one MTP. In groups of parallel cultures with different filling volumes the oxygen transfer rate (OTR, black lines) and the dissolved oxygen tension (DOT, blue lines) (a–d, m–p) are monitored. The number of parallel cultures is given in brackets behind the filling volume. Also scattered light at 650 nm (green lines) and YFP fluorescence (λ_ex_ = 480 nm; λ_em_ = 522 nm, orange lines) (i–l, u–x) are presented. The average maximum oxygen transfer capacity (OTR_max_) is highlighted by a black horizontal dotted line for each filling volume. Based on OTR and DOT, the volumetric oxygen transfer coefficient (k_L_a, red lines, Eq. 1) and the corresponding errors (light red shaded area, Eq. 2) were calculated (e–h, q–t) for OTR > 5 mmol L^−1^ h^−1^. Cultivation conditions: 0.1 g L^−1^ infrared oxygen-sensitive nanoparticles; 48 round deep-well MTP without optodes, n = 1000 rpm, shaking diameter d_0_ = 3 mm, 37 °C.

**Additional file 4: Figure S4.** Combined µRAMOS and BioLector setup. The MTP is placed on the shaker plate. The µRAMOS cover was fixed on top of the MTP to achieve equal gassing in each well and allow measurements of the oxygen transfer rate (OTR). Below the MTP an optical sensor for dissolved oxygen tension (DOT) measurement and an optical fiber bundle for scattered light and fluorescence measurements is displaced from well to well by means of an X–Y-positioning device.
