# Supplementary figures and images for: Quasi-continuous parallel online scattered light, fluorescence and dissolved oxygen tension measurement combined with monitoring of the oxygen transfer rate in each well of a shaken microtiter plate
Source: Microb Cell Fact. 2016 Dec 3;15:206. doi: 10.1186/s12934-016-0608-2 (PMC5135821; doi:10.1186/s12934-016-0608-2)

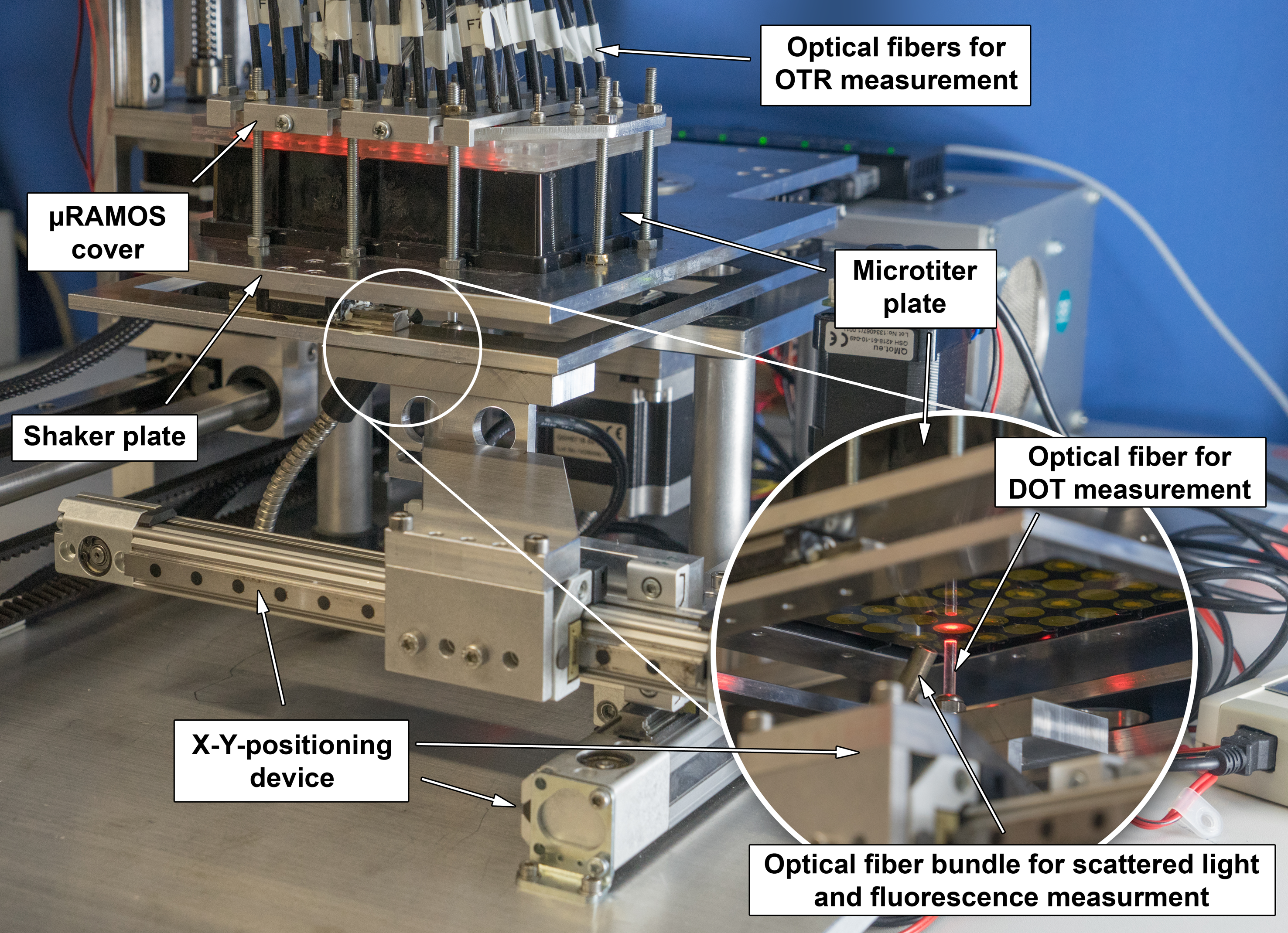

Supplement: Supplementary file 4 — Additional file 4: Figure S4. Combined µRAMOS and BioLector setup. The MTP is placed on the shaker plate. The µRAMOS cover was fixed on top of the MTP to achieve equal gassing in each well and allow measurements of the oxygen transfer rate (OTR). Below the MTP an optical sensor for dissolved oxygen tension (DOT) measurement and an optical fiber bundle for scattered light and fluorescence measurements is displaced from well to well by means of an X–Y-positioning device. [file 12934_2016_608_MOESM4_ESM.tif]
